# A Hybrid Service Selection and Composition for Cloud Computing Using the Adaptive Penalty Function in Genetic and Artificial Bee Colony Algorithm

**DOI:** 10.3390/s22134873

**Published:** 2022-06-28

**Authors:** Seyed Salar Sefati, Simona Halunga

**Affiliations:** Faculty of Electronics, Telecommunications and Information Technology, University Politehnica of Bucharest, 060042 București, Romania; simona.halunga@upb.ro

**Keywords:** service composition, cloud computing, quality of service (QoS), adaptive penalty function genetic algorithm, artificial bee colony

## Abstract

The rapid development of Cloud Computing (CC) has led to the release of many services in the cloud environment. Service composition awareness of Quality of Service (QoS) is a significant challenge in CC. A single service in the cloud environment cannot respond to the complex requests and diverse requirements of the real world. In some cases, one service cannot fulfill the user’s needs, so it is necessary to combine different services to meet these requirements. Many available services provide an enormous QoS and selecting or composing those combined services is called an Np-hard optimization problem. One of the significant challenges in CC is integrating existing services to meet the intricate necessities of different types of users. Due to NP-hard complexity of service composition, many metaheuristic algorithms have been used so far. This article presents the Artificial Bee Colony and Genetic Algorithm (ABCGA) as a metaheuristic algorithm to achieve the desired goals. If the fitness function of the services selected by the Genetic Algorithm (GA) is suitable, a set of services is further introduced for the Artificial Bee Colony (ABC) algorithm to choose the appropriate service from, according to each user’s needs. The proposed solution is evaluated through experiments using Cloud SIM simulation, and the numerical results prove the efficiency of the proposed method with respect to reliability, availability, and cost.

## 1. Introduction

In the last several years, Cloud Computing (CC) has become very popular, due to the benefits offered by cloud services in terms of the facilities provided by its hardware and software, combined with the low cost of this equipment [1]. In addition, cloud users do not need to have computer knowledge or Information Technology (IT) proficiency to use these services [2]. In fact, clients only pay for the services, and are not involved in technical issues and other practical complexities necessary for the provision of the services [3]. CC is being redesigned to provide services similar to traditional utilities like electricity, water, gas, and telephone, offering them at lower costs [4]. The CC architecture provides three primary services according to the customers’ needs. Software as a Service (SaaS) [5] offers users access to different applications as a service in the initial stage. Next, Platform as a Service (PaaS) provides the platform for constructing more complex applications, like Google Engine [6]. Lastly, Infrastructure as a Service (IaaS) offers a platform for developing and deploying virtual machines and storage facilities [7]. CC aims to develop an intelligent, robust, and secure network to access their services, based on competitive costs and Quality of Service (QoS) [8]. A group of cloud providers, such as private or public clouds, may assess the QoS of the cloud while performing a set of relevant services.

In CC, there are two types of resource service requesters: (1) a single service request, that can be completed by a single resource service, and (2) a multiple service request, that can be completed by numerous services in a given order. Offering one optimal service is straightforward [9], but selecting a set of many services simultaneously can be more challenging [10,11]. In many circumstances, users’ demands may only be fulfilled by aggregating and combining several resources and services [12], in a process known as service composition. One of the significant challenges in CC is integrating existing services to meet the intricate necessities of different types of users [13]. The cloud providers must combine different available services in order to fulfill the users’ requests. Service composition is an Np-hard issue [14], and one single service cannot respond to a large and complex request [15]. These factors determined the necessity of using services composition in order to build larger services with superior functionalities [16]. Service composition will receive broad acceptance only when consumers are confident that the offered services are reliable. One of the most important concerns in CC is creating cloud services based on trust value. Availability [17], responsibility [18], success [19], ability [20], reliability [21], and cost [22] are traits that build trust.

To tackle Np-hard issues, metaheuristic algorithms have to identify near-optimal solutions with the best possible performances [23]. The fundamental purpose of this research is to offer a unique approach for choosing the most appropriate service that responds to users’ requests in terms of QoS. In this paper, an attempt is made to solve the abovementioned issue with the help of an adaptive penalty function developed using the Genetic Algorithm (GA) and Artificial Bee Colony (ABC) algorithm. First, the GA finds the appropriate services, according to the fitness function. Then, these services are introduced to the ABC algorithm to combine them, according to a predetermined evaluation criterion, based on the user’s set of QoS. Briefly, the contributions of the current article are:

the optimal collection of services has been determined, that are contingent on the QoS criteria upon which the services are constructed, in order to fulfill the user’s objective;
the response time and the cost-of-service choices have been decreased and, subsequently, this raises the speed of service composition;
the power consumption has been reduced in comparison to another metaheuristic algorithm presented in the literature.

## 2. Related Work

This part assesses some of the most relevant papers previously published in this area and explains their contributions. The articles reviewed in this section have examined the composition of the service using various methods. A selection technique involves a set of service composition instructions, based on the available information in the system at runtime.

### 2.1. Deterministic Methods

Deterministic methods pursue a strict methodology to describe activity arrangement in resolving task disagreements, which are often said to be incorrect or uncertain and, hence, get locked in a local optimum [24]. Deterministic techniques are effective for solving minor size difficulties. A deterministic approach may be described as an automaton that performs discrete transitions between different states. In service composition, applying a deterministic method may result in the ability to forecast network performance and QoS. The availability of a network and its recursive behavior is a critical parameter in CC, which may be evaluated using a deterministic approach.

Yaghoubi and Maroosi [25] provide a service composition approach that concerns the Service Level Agreement (SLA) constraints, using an Improved Multi-verse Optimization Algorithm (IMVO). In this proposed algorithm, the distance traveled is defined as a weighted summation of the QoS attributes determined by the user’s preferences and the path of the search space close to the top solution is used in order to identify a superior one. The traveling distance in this algorithm is an essential parameter because it directly decreases the repetition. When repetition for finding suitable services fails in this algorithm, a new, improved algorithm is developed. This method brings higher availability and reliability, but the authors did not pay attention to energy consumption and the algorithm’s complexity. Song, Wang [26] presented an approach for predicting the trustworthiness of service compositions that are based on a Bayes reliability evaluation. To assess the productivity and precision of the strategies, their paper focused on fully utilizing the previous information provided by the services, and developed a Markov Model (MM) to emulate the compositions framework and each service’s reliability. Their method brings higher reliability, but the authors did not pay attention to energy consumption and cost. Jia, Lu [27] proposed a strategy based on Hidden Markov Models (HMMs) to autonomously identify service composition process faults. The analytical technique combines historical information regarding the process into a model-based analysis system to overcome the restrictions due to incomplete process model and limited historical process data. Additionally, it provides a diagnostic system design that simplifies the investigative procedure, expands capabilities, and ensures services’ privacy. This method guarantees fault tolerance and brings high reliability, but suffers scalability, inconsistency in comparison issues and it is difficult to revise comparison in the method. Kumar, Kumari [28] developed a novel methodology for service composition, that increases the reliability of service-oriented systems, named the Topsis algorithm. This technique is used to select reliable volunteer services in the initial phase, and the algorithm is solved using a prioritizing strategy based on service QoS similarity. The Topsis algorithm for service composition has been developed on the grounds of an order preference and resemblance to obtain an ideal answer. Their approach facilitates different users to access diverse options of services based on QoS criteria; the criteria are ranked and prioritized, based on their QoS performances, and then the decision is made, based on a multi-criteria methodology. This method brings low response time and high reliability, but suffers from high energy consumption and also high computational complexity.

### 2.2. Metaheuristic Methods

In various engineering and scientific applications, detecting the maximum and minimum values is a critical task. To address specific issues, efficient analytical-based methods are available. However, no practical techniques for discrete and combinatoric optimization issues have been developed yet. Heuristic approaches employ empirical methods or approximations that do not guarantee an optimal general solution but are sufficient to achieve approximate solutions for given specific problems. This technique is satisfactory in a limited time frame and can also significantly speed up the optimization process [29].

Zhang, Yang [30] proposed a service composition system based on a flower pollination algorithm. This approach is used to classify the services based on their inconsistencies with respect to the QoS parameters. A novel fuzzy-based quality-of-service-aware mathematical model is used to account for preferences, by weighting distinct subtasks in order to determine a global fuzzy QoS. This algorithm achieves good results with respect to efficiency in reliability and availability, but the authors do not take into account latency and scalability issues. Alamri [31] suggested another technique to determine the best service composition route by expressing an optimization problem with QoS constraints and solving it using an Ant Colony Optimization (ACO) algorithm. The ACO algorithm was used in their paper to compose the services and select the optimal configuration for different devices. The ACO algorithm selects the best service composition strategy, while the ants control the most appropriate composition path between the services for each user. The algorithm proposed achieves a low encoding time, but, nevertheless, the mentioned technique suffers from low load balancing and availability. Jatoth, Gangadharan [32] present a unique Optimal Fitness Aware Cloud Service Composition (OFASC) based on an Adaptive Genotype Evolution-based Genetic Algorithm (AGEGA) to cope with varying QoS boundaries and to provide service arrangements that fit the best with the user demands. The suggested algorithm satisfies the varying QoS bounds and service composition’s connection restrictions. The empirical investigation demonstrated that their approach reached superior performance in terms of process convergence speed and computational complexity, but it had the disadvantage of high energy consumption. Liu, Wang [33] introduced the unique hybrid approach to solve Manufacturing Service Composition (MSC) recommendations. At first, a Clustering-based Collaborative Filtering (CCF) procedure is used to determine customer priority restrictions and, after that, an enhanced personalization-oriented third generation algorithm is presented. The method proposed in their paper proved to attain a good efficiency in energy consumption, but the reliability of the overall process was reduced.

### 2.3. Service Recommendation Methods

The number of cloud services in manufacturing processes is rapidly increasing, and online service platforms have become more widespread [34]. Large volumes of customer-related data are stored in multiple service platforms that include, if possible, preference information. Service recommendation technology may quantify consumer preferences more precisely by exploring this data, creating clustering comparable groups of customers and perform attribute analysis of diverse user activity data [35]. Furthermore, big data increase the versatility of service recommendation expertise and allow the parameters of algorithms to be altered dynamically, depending on change in customer preferences. Collaborative data processing, context-oriented recommendation, and heterogeneous networks are now some of the most common service recommendation technologies [36]. One of the most well-known customized recommendation procedures is collaborative filtering, that dives into users’ preferences for various patterns, based on their general comportment data (e.g., purchase history, service evaluation, search history, etc.).

Li, Ma [37] presented a trust-conscious service trading system for efficiently matching numerous cloud services to meet varied user demands. T-broker produces excellent outcomes in many common scenarios, and the suggested mechanism is strong enough to manage a wide range of service resources, but, unfortunately, suffers from high energy consumption. Sefati and Navimipour [14] suggested an efficient method based on an HMM for addressing the service composition problem while improving QoS. The model was trained to forecast the QoS parameters, and a Viterbi algorithm was used to improve the transition matrices. They used the ACO method to discover a viable route. This method brings high reliability and availability, but it also suffers from high energy consumption and the high complexity of the algorithm. Kuang, Yu [38] presented a customized QoS prediction technique based on users’ reputations and positioning knowledgeable collaborative filtering for Cyber-Physical Systems (CPS) services, as well as a framework for QoS prediction for those services. Their method first estimates the user reputation using the Dirichlet probability to detect untrustworthy users and handles their faulty data, and, then, it delves the geographic location into three layers to enhance the user and service similarity calculations. This method has good efficiency in scalability and performance, but it suffers from a long convergence time issue. Su, Xiao [39] employ a beta reputation system to cluster users and to compute their information. Then, a group of similar trustworthy users is selected based on the determined user reputation and similarity. Finally, by clustering the services, they find similar services and create predictions for active users based on the QoS and similar services. This solution has good efficiency with respect to low response time and high throughput, but it suffers from a low load balancing issue. Li, Ma [40] concentrated on the trust computing requirements of multiple-cloud collaboration services and created Data-driven and Feedback-Enhanced Trust (DFET) computing. In this case, a distributed soft agent-based trust-conscious service monitoring system is suggested as a middleware for multi-cloud trust computing. This method is suited for service-oriented cloud applications and numerous critical service indications in trust computing. More importantly, they suggest an upgraded hierarchical feedback mechanism based on the fundamental interaction between users, monitors, and service providers, which may substantially minimize the networking risk while enhancing system reliability.

### 2.4. Comparison and Overview

Deterministic algorithms have proved to have good performance in availability and response time. Besides, the energy consumption of their algorithms, compared with ones from other categories, is lower, but they have proved to be unsuitable for some issues, such as the Np-hard problem. Metaheuristic algorithms are mostly composed of heuristic algorithms that can be more responsive to Np-hard problems. In the metaheuristic technique, these algorithms have high complexity and high energy consumption in comparison with other methods. However, these algorithms have proved to have good scalability in service composition. According to a related literature study, many articles evaluated the algorithms in terms of cost, duration of convergence rate, and reliability. Still, most of the proposed methods have not been tested in real environments. According to the literature review, the vast majority of service recommendation technologies increase the quantification accuracy of consumer preferences and ensure suggestion outcomes better follow changes in customer demands. However, most of the particular service solutions presented are limited. The described technologies develop practical solutions that face the challenges of flexible and diversified composite services. Their results are readily invalidated when customer-related behavior data is insufficient. Consequently, multi-attribute decisions incorporate both the QoS objective aspects of fabrication services as well as the preference attributes of consumers. This technique is a more successful approach than unilateral subjective decision-making in obtaining recommendation outcomes.

## 3. Motivation

**Figure 1:**
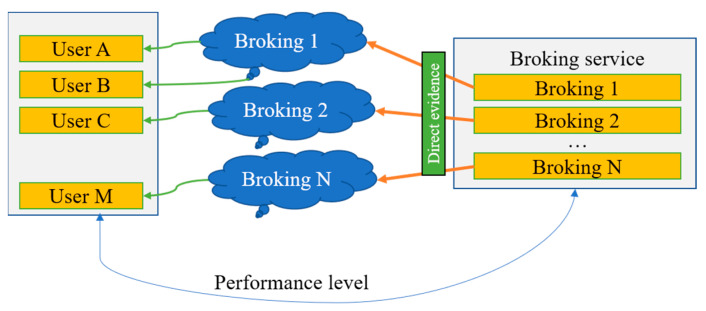
Existing brokering scenario without user feedback.

RESERVOIR [41], PCMONS [42], RightScale [43], SpotCloud Aeolus [40], and OPTIMIS [44] are examples of CC that have recently emerged as potential solutions for providing superior service to customers. The development of cloud brokers works as an intermediate among cloud suppliers and clients to settle and transfer resources [45]. Unfortunately, except for OPTIMIS, the majority of these CC do not offer trust management abilities for clients [46]. In Figure 1, users connect to the providers to use the cloud services according to the needs of QoS. Brokering services provide data to the cloud as storage and select the service according to the proposed algorithm. All the M users that access the cloud simultaneously can benefit from the cloud brokering in high QoS. Managing and planning the existing resources with high trustworthiness requires an accurate technique. Evaluating and forecasting consumption patterns of computational resources is problematic because it can change dynamically over time. This research aims to develop a service composition algorithm that efficiently matches the existing computer resources to various user demands. This subject has attracted the interest of different researchers, but their methodologies have not been able to significantly advance created notions in earlier trust models [47].

### 3.1. Problem Statement

**Figure 2:**
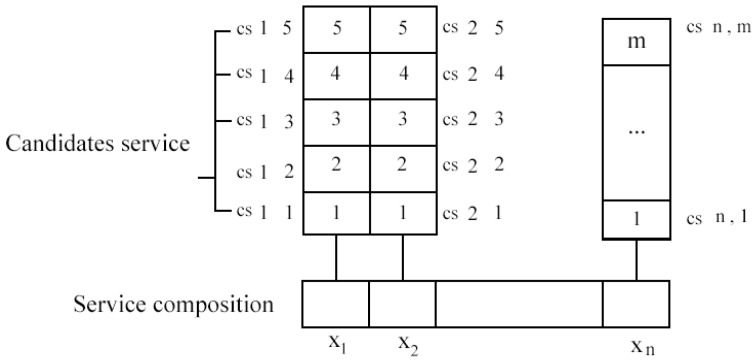
Service composition method.

The service composition aims to discover the most suitable set of Cloud-based Manufacturing Services (CMSs) from a pool of services for delivering enhanced user experience while meeting the QoS limitations [48]. In order to increase efficiency, a manufactured product has to be broken down into multiple sub-products. QoS-aware composition targets discovering a set of candidate services with comparable functionalities to improve users’ satisfaction and the overall QoS quality [49]. Figure 2 shows the formal definition of service composition, where it has been assumed that, from the total of *m* candidate services that are available in the cloud, *n* services, $\left(X_{1},\;X_{2}\;\cdots \;X_{n}\right)$ have to be combined with each other in order to achieve the targeted QoS and configured to meet the user’s needs. There are many different modes of combining a set of services, but it is excessively time-consuming to consider all possible ways and select the optimal method. This paper solved the service combining issue by using Artificial Bee Colony and Genetic Algorithm (ABCGA).

As has been previously mentioned, service composition techniques combine several separate services to achieve the highest QoS and offer the best services for users [50]. The QoS offered by different services is constantly changing, therefore the candidate services’ criteria need to change over time. The Service-Level Agreement (SLA) is a sort of understanding between consumers and cloud service providers that meets the QoS standards. Recommendation services are one of the cloud-based intelligent strategies for addressing the challenge of locating acceptable services in order to answer all the users’ needs. The recommendation system can suggest the best services for a set of users. In contrast, the services in the cloud have a lot of QoS requirements, so finding suitable services is a challenging task. A candidate service is recommended to each target user depending on the profile. For instance, Amazon’s Kindle Bookstore employs suggested technology for books and CDs, while Netflix.com uses it for movies, with over 17,000 films. They are well-positioned in the market.

### 3.2. The QoS-Aware Service Composition

The scope of QoS-aware service composition is to select the optimal execution plan to maximize the end-to end QoS of the service composition. In most cases, besides selecting the optimal execution plan that takes exponential time and costs, new approaches are satisfied with finding a nearly optimal solution, even when simplifications are used. We will present several definitions that might help define the issue more clearly below.

**Definition** **1.** *Assuming that there are two services $S_{1}$, $S_{2}$ ∈ S, connected to a set, (Q) of QoS criteria, we say that $S_{1}$ dominates $S_{2}$, denoted as $S_{1}$ < $S_{2}$, if $S_{1}$ superior than $S_{2}$ in all parameters in (Q) and superior in at least one parameter in (Q), i.e., ∀k ∈ [1, |Q|]: $q_{k}$ ($S_{1}$) ≤ $q_{k}$ ($S_{2}$) and ∃k ∈ [1, |Q|]: $q_{k}$ ($S_{1}$) < $q_{k}$ ($S_{2}$), where the notation $q_{n}$ ($S_{m}$) represent the $n_{th}$ QoS attribute of the $m_{th}$ service [3].*

**Definition** **2.** *A set of superior services (SL_S_) is described as a collection of services in (S) that are not dominated by another service. i.e., SL_S_ = ($S_{i}$ ∈ **S**|$∄S_{k}$ ∈ S: $S_{k}<S_{i})$ [3].*

The aim of the service composition is to discover the most suitable set of CMSs from a pool of services for delivering enhanced user experience while meeting the QoS limitations. Sometimes, we may encounter challenges in a broad range of applications in cloud manufacturing, when finding a service composition is a difficult task. Assuming that *N* is number of services involved in the combination process, (***S***) = ($T_{1}$, $T_{2}$, $T_{3}$, …, $T_{N}$) represents a particular combination of tasks, *M* is the number of the candidate services available for each abstract service and ($C_{u}$) = ($C_{u1}$, $C_{2u}$, $C_{3u}$, …, $C_{Mu}$) is the number of existing services. This issue can be mathematically formulated as follows.

(1) $$\mathrm{Maximize}\;\sum_{k=1}^{D}w_{k}\;f_{k}\;(\{\sum_{i=1}^{N}\;\sum_{j=1}^{M}\;S_{ij}C_{u}\})$$

(2) $$\mathrm{Subject}\;\mathrm{to}\;f_{k}(\{\sum_{i=1}^{N}\sum_{j=1}^{M}S_{ij}\;q_{ijk}\})$$

(3) $$\sum_{k=1}^{D}w_{k}=1,\;w_{ij}\in \{0,1\}$$

(4) $$\sum_{k=1}^{D}S_{ij}=1$$

(5) i=1,2,…,N,j=1,2,…,M

In the equations above, we denoted the weight of the QoS characteristic by $w_{k}$. The aggregation function, denoted by $f_{k}$, is used to evaluate the mixed rate of *k* quality. $S_{ij}$ shows the specific service selection, the particular service *j* being chosen for an abstract service *i.* $q_{ijk}$ shows the QoS of the selected service. $w_{ij}$ determines whether the candidate of cloud-based service CMSj is selected or not. When the combinatorial is optimized, it must meet the global QoS attribute restriction and the maximum value, as stated in Equation (1).

**Figure 3:**
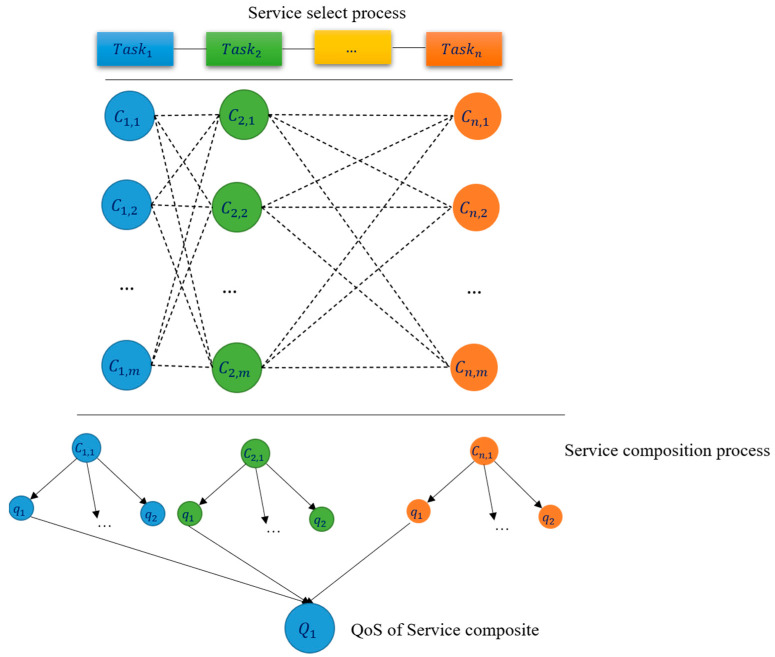
An intuitive description of QoS-aware method for service composition.

The method of creating services using the integer coding strategy is shown in Figure 3. The sequential combination is (***S***) = ($Task_{1}$, $Task_{2}$, …, $Task_{N})$, in which each task or candidate service is associated to an abstract service. Each service has *N* concrete tasks, each of them set such to satisfy different QoS parameters. One particular service is chosen from many available services, and all of the services have a specific duty in the cloud environment. The optimal value is determined by combining those physical services to maximize Equation (1) with the restriction given in Equation (2). Each service sets a unique number in this combination of integer-coded services. For example, in Figure 3, $Task_{1}$ candidate service has been chosen $C_{11}$, $Task_{2}$ been chosen $C_{2,1}$ in the integer code, and at the end the sum of these integer codes shows the service composition.

### 3.3. Objective Attributes of QoS

The QoS measures non-functional aspects of different services, such as time delay in response, accessibility, and cost. The components, such as response time, energy consumption, and latency must be reduced, while availability and reliability must be enhanced. Table 1 describes the QoS parameters in CC.

The **response time** is the capability of a service to fulfill the necessary functions under determined circumstances in a given time interval, and it can be mathematically described by Equation (6) [14].

(6) $$RA_{Rk}=\frac{RES_{k}}{REC_{k}},\;k=\bar{1,M}$$

where, $RES_{K},\;k=\bar{1,M}$ represents the number of tasks sent to a certain service, $R_{K}$, that were performed within a specified period, typically of several tasks in milliseconds, while $REC_{K}$ indicates the total number of requests.

**The availability** refers to the capacity of a service to be available and operable at any time, and it evaluates if a certain service may be used for the whole duration of a given task. Mathematically, it can be described by Equation (7).

(7) $$AV_{R_{k}}=\frac{A_{k}}{N_{k}},k=\bar{1,M}$$

where, $AV_{R_{k}}$ is the availability of resource $R_{k}$, $N_{k}$ shows the total number of tasks submitted to $R_{k}$ while $A_{k}$ defines the number of jobs accepted by the $R_{k}$.

The **Cost** is the quantity of funds necessary to satisfy the requirements of a virtual service depending on the total amount of memory used, processing time, and bandwidth consumed. It may be mathematically evaluated using Equation (8).

(8) $$Cost=\sum_{i=1}^{K}\left(C_{i}*T_{i}\right)$$

where *K* is the size of the service, $C_{i}$ is the number of assigned users’ requests and $T_{i}$ is the time for which the user can use the services.

**Reliability** refers to certain hardware or software equipment’s capacity to complete a job in a specific time, depending on system requirements, Equation (9).

(9) $$RE_{RK}=\frac{C_{k}}{A_{k}},\;k=\bar{1,M}$$

where $A_{k}$ is the number of jobs that have been accepted by a certain resource $R_{k}$ and $C_{k}$ the number of jobs successfully completed by this same resource.

The **energy consumption** represents the quantity of energy necessary to fulfill a certain task, and it is usually evaluated via the processing time or processor usage.

(10) $$U_{i}=\sum_{j=1}^{n}u_{i,j}$$

where *n* is the number of tasks running at that time and $u_{i,j}$ is amount of the resource used.

**Stability.** In a dynamic Cloud Manufacturing (CMF) system, different services may change from one time instant to another. If the QoS of a service is unstable and values fluctuate, the consumers cannot accept this situation; thus, the fluctuating of QoS services will pose a danger to service selection and composition. The QoS stability (Sta) of service $S_{i}$ may be characterized based on standard deviation as shown in Equations (11)–(13).

(11) $$Sta(S_{i})=\frac{1}{p}\sum_{q=1}^{p}Sta_{q}\;(S_{i,q})$$

(12) $$Sta_{q}(S_{i,q})=1-\frac{1}{S_{i,q}}\sqrt{\frac{1}{M}}\sum_{t=1}^{M}(S_{i,q}^{t}-S_{i,q}{)}^{2}$$

(13) $$S_{i,q}=\frac{1}{M}\cdot \sum_{t=1}^{M}S_{i,q}^{t}$$

*Sta*$\left(S_{i}\right)$ denotes the total QoS stability of service $S_{i}$ and ($S_{i,q}$) is the stability of a certain attribute *q* of the service $S_{i}$ in the time period of interest, $S_{iq}^{t}$ denotes the attribute *q* value of service $S_{i}$ at the *t* moment, while M indicates the number of jobs sent to the service in *t* time.

Since the various QoS characteristics have distinct measurements and optimization features, normalization is essential in order to guarantee that each QoS goal attribute has the same assessment standard and logic. Therefore, they should be standardized. Positive and negative QoS features are divided into two groups. For positive characteristics, such as reliability or availability, the higher the numerical value the better the quality. In contrast, for negative factors, such as response time or cost, the lower the numerical value the better the quality. To prevent inaccurate calculation of various QoS measurement criteria, attribute values should be normalized, so that all QoS characteristics are evaluated on the same scale. Therefore, to normalize these characteristics on a standard scale, the positive attributes of Equation (14) are used, and for the negative factors, Equation (15) is used:

(14) $$q_{s_{ij}}^{l}=\left\{ \begin{array}{ll} \frac{q_{s_{ij}}^{l}-Min(q_{s_{ij}}^{l})}{Max(q_{s_{ij}}^{l})-Min(q_{s_{ij}}^{l})} & ,\;Max(q_{s_{ij}}^{l})-Min(q_{s_{ij}}^{l})\neq 1\\ 0 & ,\;Max(q_{s_{ij}}^{l})-Min(q_{s_{ij}}^{l})=1 \end{array}\right.$$

(15) $$q_{s_{ij}}^{l}=\left\{ \begin{array}{ll} \frac{Max(q_{s_{ij}}^{l})-q_{s_{ij}}^{l}}{Max(q_{s_{ij}}^{l})-Min(q_{s_{ij}}^{l})} & ,\;Max(q_{s_{ij}}^{l})-Min(q_{s_{ij}}^{l})\neq 1\\ 1 & ,\;Max(q_{s_{ij}}^{l})-Min(q_{s_{ij}}^{l})=1 \end{array}\right.$$

$q_{S_{ij}}^{l}$ represents to the l quality of the j service from the i service while $Max\left(q_{S_{ij}}^{l}\right)$ and $Min\left(q_{S_{ij}}^{l}\right)$ are the extreme values of the l for the i service.

The critical objective of service composition is to provide QoS services that adhere to user-defined limitations and improve a fitness function, which should be to optimize the rates of the QoS parameters. Positive and negative parameters have an inverse relationship with their evaluation function. The competence function should maximize the importance of the built-in composite service’s QoS parameters. Positive and negative parameters have the opposite tendency and have the opposite influence on the evaluation function.

(16) $$QoS(WS)=Min\left(\sum_{i=1}^{D}w_{i}\cdot QoS(f_{i})\right)$$

where $f_{i}$ represents the quality value of each parameter from the service *S_i_*, and $w_{i}$ represents the user’s desired weight for that quality parameter in the overall QoS, and *D* is the number of service dimensions for a workflow.

### 3.4. ABCGA Algorithm

In this paper, the ABCGA algorithm is applied to solve the service composition problem in cloud computing. The correspondence between the ABC and GA algorithms in service composition is shown in Table 2 and Table 3. Since we wanted to preserve the notations from the original algorithms, in the following table, the correspondence between those notations and their correspondent service selection and composition for cloud computing is provided.

An intelligent algorithm has many benefits, but there are often several disadvantages. For example, GA considers the values of the target function immediately as search information, and, thus, demonstrates archiving, robust resilience and high search rate. Moreover, GA has proved efficient for solving large-scale challenges. However, GA’s search performances are highly influenced by reduced local search capacity and, thus, GA is easily localized. A Genetic algorithm is a type of meta-algorithm based on biological theory that is random and intelligent. The process begins with generating an entirely random collection of entities and random actions on prior generations that will cause future generations. From each generation, the best entity is selected. If the customer’s requirements are met, that entity is introduced as the answer to the problem, and the algorithm ends. Otherwise, another set of new entities is established, and the process continues until it achieves the customer requirements. If an appropriate entity is not found, the algorithm terminates. When it reaches the specified maximum number of iterations and introduces the last entity obtained from those iterations, it is presented as the best answer to the problem. Each chromosome is made up of several genes and random selection and combinations of genes create new chromosomes. One of the factors in GA is called a crossover, and the random selection of genes from chromosomes is called a mutation.

The idea of the ABC algorithm starts from an example in nature, where the bees can spread in different locations and over long distances to obtain food sources. Pollen can be collected with little effort in areas where there is a large amount available. Usually, if the region has a high pollen quantity, there is a high probability that many bees will visit these areas. Worker bees seek to find a new food source based on their already known ones. Using the information supplied by worker bees, the observer bee is continuously searching for new food sources. Finally, when the pollen source is used up, the bees will randomly seek new areas for food supply. The ABC algorithm is a universal search procedure that can effectively avoid local optima but suffers from long search times and slow convergence.

**Step1:** In the first stage, the initial population is set for the GA and it calculates the chromosomes’ suitability. It is a sequence of numbers that can be considered one of the answers to the problem, and the algorithm is seeking to determine the best values for each of the genes in order to reach the optimal point. Therefore, the chromosome arises from the parameters of this algorithm. The fitness function must be able to determine the best combination of services. For this function, the quantitative and qualitative characteristics of the selected services should be examined. According to the data set used, and its properties, the objective function is defined as the weighted average of the Equation (16) properties.

**Step2:** In order to improve outcomes from one generation to another, the chromosomes should be selected. Equation (17) indicates the probability of selecting a chromosome, *P*(*x*), where f(x) represents the quality value of each parameter and (Σ*f*) shows the quality of value of all of them. In Equation (17), the ratio of the fitness function of one chromosome to all chromosomes is calculated. The more significant is the number obtained, the more likely the chromosome will be selected.

(17) P(x)=f(x)⋅∑f(all)

**Figure 4:**
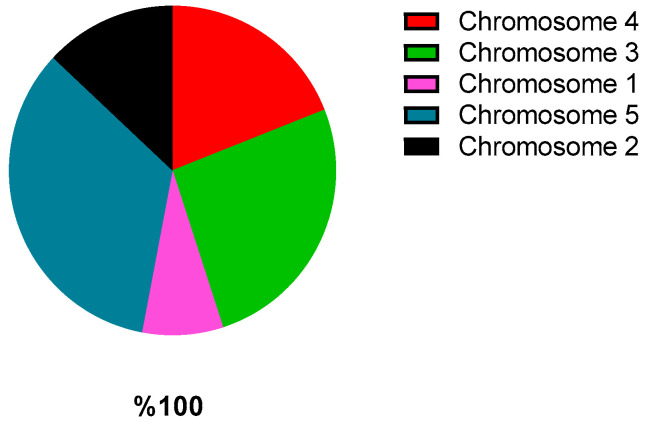
Segment of a circle of each chromosome.

Figure 4 shows an example of a pie-type diagram in which a segment of a circle is assigned to each chromosome according to its fitness.

**Step3:** The production of child chromosomes from parent chromosomes is known as crossover. In this study, the weighted average intersection was used to combine the two parent chromosomes and produce an offspring. For each parent chromosome, a random number in the domain (0, 1) is generated. For example, if the first chromosome weighs 0.9, then the weight of the second parent will be 0.1. A mutation is a random control element in which the values of some genes on a chromosome are replaced with new ones. In this study, the chromosome is chosen randomly from the entire population. If the fitness functions of the obtained selected services are better, so this result is closer to the user requirements, GA stores them and introduces them to the ABC algorithm. Otherwise, the Adaptive Penalty Function in the Genetic Algorithm will be used. This technique uses a penalty function to support dependency constraints and interoperability between services, thus imposing fines for the impossible solutions. In the penalty-based genetic algorithm, such fines are used for those chromosomes that have impossible genes and have violated limitations of the interdependence and incompatibilities between services. A chromosome with many restrictions should be severely penalized. Equations (18) and (19) express the fitness and penalty functions, respectively.


(18) $$\left(x)=0.5+(0.5*F_{obj}(x))+P(x\right)$$

(19) $$P(x)=\left\{ \begin{array}{l} 0,if\;v(x)=0;\\ -0.5-\frac{v(x)}{v_{\max}} \end{array}\right.\;\mathrm{Otherwise}$$

In the first equation $F_{obj}$ is the function that evaluates and reflects the qualitative properties of the fit function for the *x* chromosome. In addition, *P*(*x*) is the penalty calculated and attributed to the *x* chromosome. In the second equation, v(x) represents the total number of constraints violated by the *X* chromosome, while $v_{max}$ is the maximum number of cases of restriction violations. Therefore, when v(x) is zero, this shows that chromosome *x* has not violated the constraint. According to Equation (18) if chromosome x corresponds to a resolvable solution, the penalty will be zero. Otherwise, it calculates the amount of fine imposed on the x chromosome, as an impossible solution, ensuring thus that the more restrictions an impossible solution violates, the higher will be the fine applied.

**Step4:** After GA finds the most suitable QoS service, the ABC algorithm starts to combine services to satisfy the user’s need.

**Step5:** Regarding the search for a service location, the ABC algorithm looks for the best possible answer in the search space, creates a new search location for each service, and then moves on, as suggested in Equation (20)

(20) $$Q_{ik}=Q_{ij}+\phi \cdot Q_{kj}$$

$Q_{ij}$ represents the current position of the bee, $Q_{kj}$ represents the new position of the bee, φ is a random number in the range of negative one and positive one. $Q_{ik}$ represents the new position of the bees. The worker bee searches for selected services, based on the GA, and calculates the amount of nectar per flower.

**Step6:** All the population of food sources is indicated as a $x_{mi}$, and *m* shows population size. This population size shows the number of services in the CC,

(21) $$x_{mi}=l_{i}+rand(0,1)*(u_{i}-l_{i})$$

where $l_{i}$ and $u_{i}$ demonstrate the lower and upper boundaries of the parameter $x_{mi}$, respectively.

**Step7:** Employed bees search for a new food resource and in CC employed bees are trying find all of the services. $v_{m}$ shows the new food source, and $x_{mi}$ shows the nectar within the neighborhood of the food source in their memory.

(22) $$v_{mi}=x_{mi}+\phi_{mi}(x_{mi}-x_{ki})$$

where $x_{k}$ is a randomly selected food source, *i* is a randomly chosen parameter index and $\phi_{mi}$ is a random number within the range [−1, +1].

**Step8:** Since discovering the new food source (services), the fitness function of the services is evaluated; if the fitness function is desired in terms of the user requests, it is selected as the best service, otherwise it tries to find another suitable service. The fitness function is calculated for each service using the following formula. In general, the most crucial element in the ABC is the fitness function, which greatly affects the performance and efficiency of the ABC, determining whether it is successful or not.

(23) $$fit_{m}(x_{m})=\left\{ \begin{array}{ll} \frac{1}{1+f_{m}(x_{m})}, & {\mathrm{if}\;f}_{m}(x_{m})\;>\;0\\ 1+abs(f_{m}(x_{m})), & {\;\mathrm{if}\;f}_{m}(x_{m})\;<\;0 \end{array}\right.$$

where $f_{m}$ is the objective function value of the solutions.

**Step9:** If the fitness function of services is not suitable, the onlooker bees start to find new services. The onlooker bees evaluate the potential food supply in a new location, and the value of the fitness functions is determined. If the new position value is a better food supply than the previous position, then the bee forgets the last position and keeps the new one in memory. Otherwise, the bee remains in its previous position. Equation (24) describes, mathematically, how the worker bees moves:

(24) $$X_{id}^{new}=X_{id}^{old}+\phi_{id}(X_{id}^{old}-X_{id})$$

where $X_{id}$ is the *i* component of the vector that indicates the position of the onlooker bee, $X_{ik}$ indicates its previous position of the employed bee, until the bee moves towards $X_{ik}$ = ($X_{1k}$, $X_{2k}$ … $X_{n}).\;X_{id}^{old}$, indicates the previous position of the employed bee. ϕ is a random vector in the interval (1, −1). $X_{id}^{new}$ shows the new position of onlooker bees. In the upgrade phase observer bees use the status of worker bee information.

**Figure 5:**
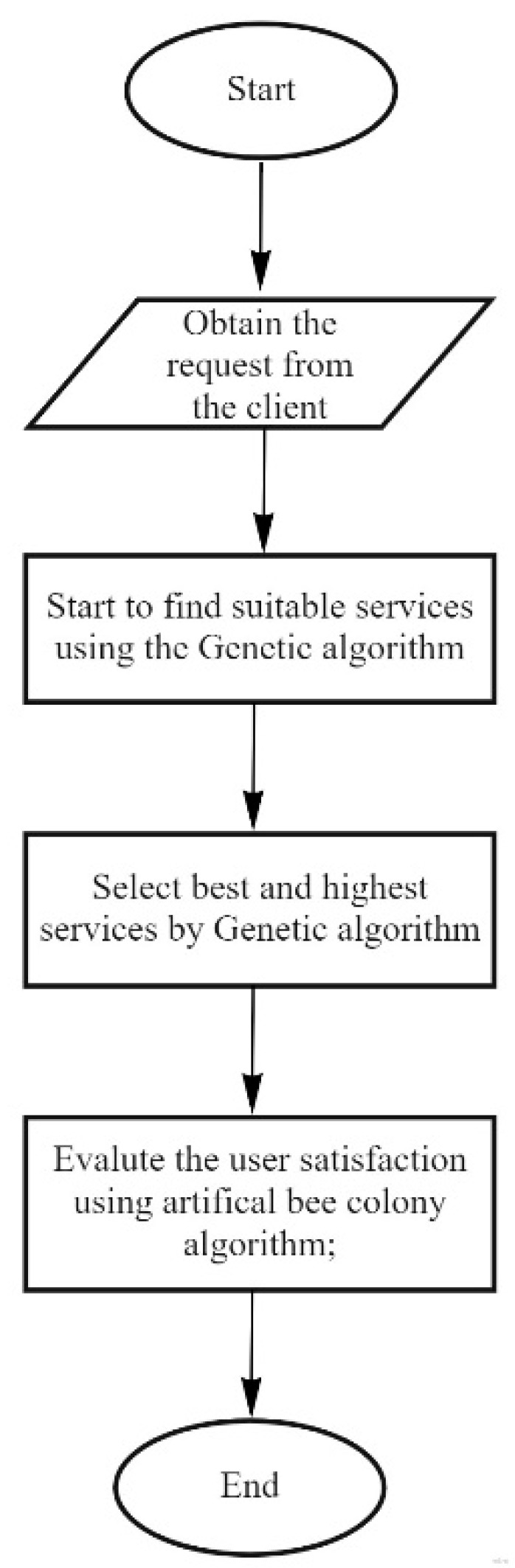
General structure of ABCGA algorithm.

In Figure 5, the proposed method is explained using a block diagram. According to this flowchart, first, we used the genetic algorithm to select the appropriate service and then, according to a user’s requests, the services are combined using the ABC algorithm. Table 4 shows the summary of notation used in the procedure and Algorithm 1 describes the ABCGA algorithm.

**Algorithm 1.** ABCGA algorithm description**Input:** Obtain the request from the client. Define a label that includes the requested service; **Output:** Suitable service Initialize C, ¥, R **While**             1.Initialize the set variables (*X*) uniformly distributed within the sampling space ¥            2.Calculate the objective function *f*(*x*)            3.Assign the number of generations to 0 (to = 0)            4.Evaluate the individuals in population            5.**If** the fitness functions the obtained optimal service            6.Go to line 16            7.**Else**            8.**While** termination function is not satisfied do
9. Apply to approach the objective function 10.φ (*x*) = *P* (*x*) ∗ *f* (*x*) 11. Where *P* (*x*) = *f* (*x*) ∗ Σ*f* (*ALL*) 12. The probability of p of each candidate service C in the cohort is calculated as: P^C^ = $\frac{\frac{1}{\varphi }*X}{\Sigma f\;\left(\mathrm{ALL}\right)\;}$ 13. Use the roulette wheel method to select, for every candidate C, the behavior to follow from the available choices. 14. Reduce each candidate C sampling interval ${¥\;}^{c}$ in its vicinity by reducing the sampling space parameters R and set of solution ${x\;}^{c}$ ${¥\;}^{c}$ = [ ${¥\;}^{c,\;lower\;QoS}$, ${¥\;}^{c\;,\;upper\;QoS}$ ] = [ ${x\;}^{c}$-|| $\frac{{¥\;}^{c,\;lower\;QoS}\;,{\;¥\;}^{c\;,\;upper\;QoS}}{2}$ || * R, ${x\;}^{c}+$|| $\frac{{¥\;}^{c,\;lower\;QoS}\;,{\;¥\;}^{c\;,\;upper\;QoS}}{2}$ || * R] Next, each candidate C will select its variable from the updated sampling interval ${¥\;}^{c}$ **15. If:** there is no significant improvement in system solution is saturated. Each candidate C should expand the sampling interval ${¥\;}^{c}$ to its original ¥ . Accept the current behavior of the group, the φ (*x*) and the associated attributes of x. Else ABC: **Initialization1. Service space exploration strategies identification.** 16. The exploration strategies in the Service space are determining **Initial service domain attributes generation** 17. $x_{m}$←Init Food Source Gen workflow, SN (α, β): m = 1, 2,…, SN. (α, β): indicates the quantity of food in the different service sets∗ **Driven employed service domain attributes (local optimization)** 18. Fit($v_{m}$)← Fitness ($fit_{us}$,$fit_{c}$,$fit_{Dc}$); m = 1,2,.., SN; $fit_{us}$: User satisfaction; $fit_{c}$: correlation ship meeting degree;$fit_{Dc}$: domain constraints satisfaction degree 19. **If** (fit ($V_{m}$) ≥ fit $(x_{m}$)) **then**
$X_{m}$←$V_{m}\;$;m = 1, 2…, SN 20. **End if** 21. **Repeat** **Local optimization–droven onlook phase** 22. $ps_{i}$←Calc selection prob (Fit(x1), Fit (x2), Fit (SN)); I = 1, 2…, SN. $x_{m}$←Select (rand (), $ps_{i}$); $V_{m}$←Neighbor exploration ($x_{m}$,Exploration strategy, η); Fit ($V_{m}$) ← Fitness $(V_{m}$), ($fit_{us}$,$fit_{c}$,$fit_{Dc}$); **If** (Fit ($V_{m}$) > Fit $\;(X_{m}$)) **then** $x_{m}$←$V_{m}$ = 1, 2… SN 23. **End if** **Store in the memory the best solution achieved so far** Global best solution← optimal selection ($x_{1}$, $x_{2}$,…, →$\;x_{m}$, Global best solution); **Arbitration Criteria.** Arbitration criteria (Max time, user satisfaction, best composite service) == true **Return Global best solution**2

## 4. The Simulation Environment

**Figure 6:**
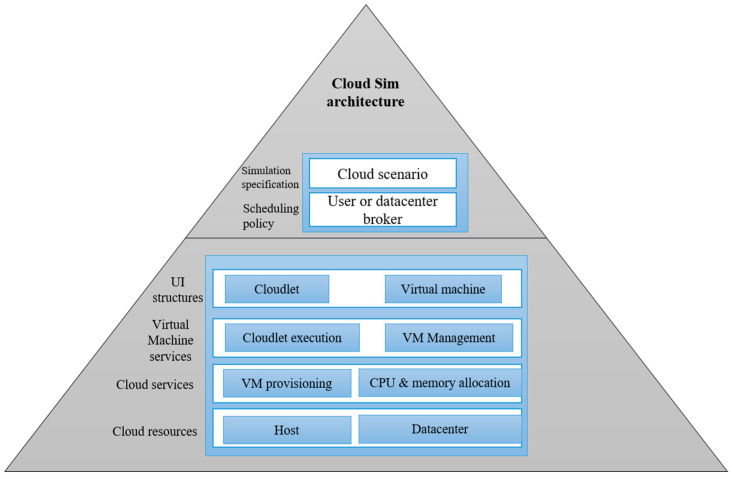
The architecture of Cloud sim.

In order to simulate the previously discussed algorithms, the Cloud SIM software was used to emulate the features, settings, and information utilized. The software ran on a computer with the following specifications: 16 GB RAM, Intel Core i7 3.2 GHz CPU. Cloud SIM is an open-source platform for simulating cloud computing services and infrastructure. It was developed by the CLOUDS Laboratory team and is totally Java-based. It is used to model and simulate a cloud computing environment and to test the hypothesis before software development, so that tests and findings may be replicated. The load distribution system attempts to enhance efficiency by transferring some processes from busy servers to underloaded servers. In the current study, the components of the Cloud SIM, including the datacenter, Virtual Machine (VM), host, and cloudlet were used to analyze and perform the simulation. The cloud SIM has lots of advantages, such as: the software is free to use; it is easy to use and scalable; the risks can be assessed early in the process; there is no need for trial-and-error methods. A block description of the components used is presented in Figure 6.

### 4.1. The Simulation Data Parameters

We used the Quality of Web Service (QWS) to generate the dataset. We generated qualifier metrics for additional qualitative features, such as practicality, safety, and adaptability, and we employed six QoS criteria with a standard data format. To make the procedure more accessible, the classification attributes for security, usability, and flexibility were labeled as low, medium, and high. Response times were divided (0.5, 2, 3 and etc.) and availability was divided in values (99.5, 99.9 and 99.999) and, also, for costs there were values (5, 20, 30, 40). After sorting based on QWS, we obtained a dataset with 50 services for the six chosen criteria. It is essential to highlight the fact that, in order to employ the proposed strategy in a real-world situation, the service provider must adjust the QoS features accordingly. Communication between the user and cloud data center was considered between 20–500 and between the user and service set between 50–400. We used Cloud SIM to emulate CC at SaaS level for testing, since this simulator allows you to create a virtual environment and manage the supply of resources necessary to meet the users’ demands.

### 4.2. Results and Discussion

**Figure 7:**
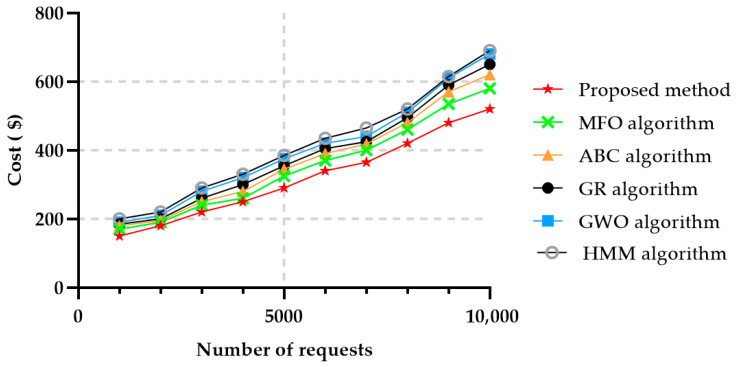
Average cost in 50 services and in different numbers of requests.

Based on the results obtained, the suggested technique was compared with Moth flame Optimization (MFO) [3], ABC [51], Greedy (GR) [52], and the Grey wolf optimization (GWO) algorithms [6], HMM [27] in the same dataset. In this paper, the cost was computed, based on the sum of the services selected, and other methods were calculated according to different criteria. The proposed method gives the user reliability to express the importance of each determining factor. Reliability is usually deployed on virtual machines (VMs) for some critical areas, such as power supply, traffic control, medical healthcare. In our method, providing the chromosomes as a service reduced the overall cost, since each chromosome and gene could find a suitable service, making it less expensive and less resource-consuming. Figure 7 shows the average cost results versus the number of requests, for the method proposed in this paper and for the other five ones mentioned above. It can be easily observed that the proposed method implied a reduced cost in comparison with the other methods, being followed by the MFO algorithm. HMM achieved an unfavorable result in the cost criteria.

**Figure 8:**
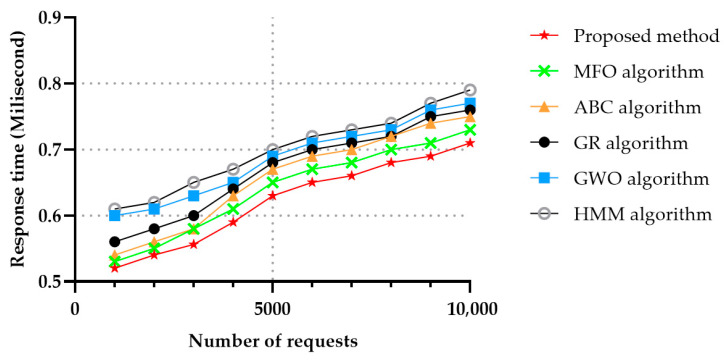
Average response time in 50 services and in different number of requests.

With respect to the response time, the cloud was trying to answer to user’s demands as fast as possible, in order to provide the desired services. The user’s needs were first analyzed and then answered using metaheuristic algorithms in our approach. In the MFO algorithm the services were found using the help of butterflies’ algorithms, which, due to low convergence speed, had a better response than the one achieved with the ABC algorithm. Since in the ABC the worker bee first seeks out the nectar of the food, then, after finding suitable food, informs the other bees, this process causes more time to be wasted. The GWO algorithm achieved similar results to the ones obtained by the ABC algorithm with respect to response time. In the GWO algorithm, alpha first seeks service, and after finding a suitable service, informs the herd. Response time elapses between sending a request and providing the data or declaring an inability to provide the data. It takes time for a memory circuit or storage device to prepare the requested data by the Central Processing Unit (CPU). Figure 8 shows the average response times in services versus the number of requests for MFO, GR, ABC and GWO algorithms, and it can be seen that the proposed method achieved the lowest response time compared to the other algorithms, followed by the MFO algorithm. HMM is a complex algorithm, therefore the response time achieved using this method was not suitable for extensive services.

**Figure 9:**
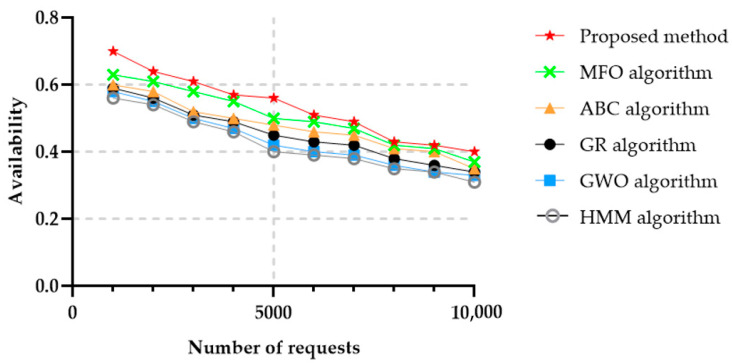
Availability in 50 services and in different number of requests.

From the cloud provider and the service consumer’s perspective, availability is one of the most critical success factors. Availability is the amount of time the equipment and the associated assets can be used by a certain service at any time instance. In general, when calculating availability, shutdown times include all scheduled times of maintenance and repairs, as well as unplanned maintenance and repair operations. The availability of the proposed method was higher than for the other algorithms it was compared with, while the GWO and GR algorithms achieved the lowest availability. Figure 9 illustrates the availability values as a function of the number of requested services.

**Figure 10:**
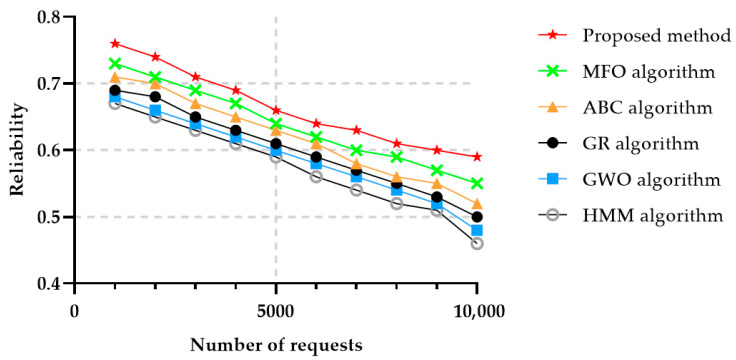
Reliability in 50 different services.

Reliability is another important parameter, as defined in Section 3.3, and evaluates whether a system or ensemble of systems offering a certain functionality item operate smoothly under specified and predetermined conditions for a specified time interval. Almost all the methods obtained slightly similar results with respect to the reliability parameter, even though the proposed method achieved a slightly higher value, as illustrated in Figure 10. HMM is a predictable algorithm, therefore the reliability of this proposed method was lower than the ones achieved by the other algorithms.

**Figure 11:**
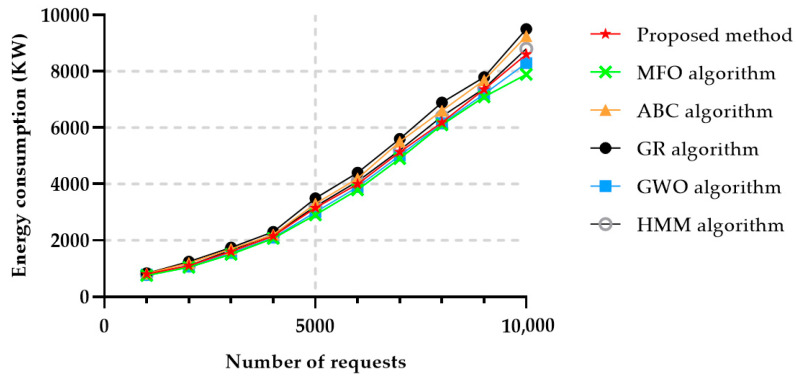
Energy consumption in 50 different services.

Figure 11 represents the energy usage as a function of the number of service requests, and it can be observed that the MFO algorithm had the best performance in terms of energy consumption, while the proposed algorithm obtained close results to the ones achieved by MFO, as long as the number of requests was relatively small. However, after 9000 requests, the energy consumption increased. The HMM algorithm also achieved good results in comparison to the ABC algorithm and GR algorithm, but the energy consumption achieved with GWO, MFO and the algorithm proposed in this paper was lower.

**Figure 12:**
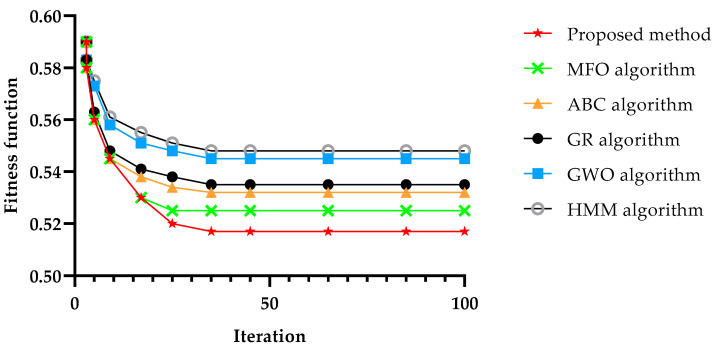
Convergence in 80 tasks and 100 rounds.

Figure 12 shows the algorithms’ convergence versus the number of service requests, and it can be observed that the proposed method converged faster than all the other methods under our study. Moreover, the convergence achieved its minimum value after around 40 iterations. Therefore, the results have been represented only till 100 iterations.

**Figure 13:**
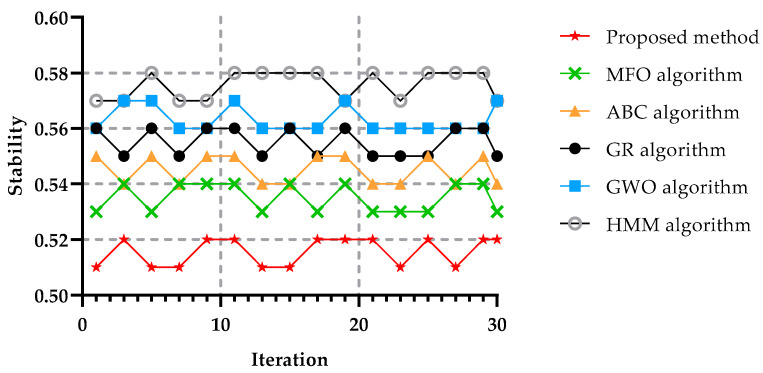
Stability in 80 tasks and 30 times the implementation of the algorithm.

The stability of an algorithm measures how good a job the algorithm does at solving problems to the achievable accuracy defined by their conditioning. Stability is often identified as a sensitivity to the disruption of input data during the process of selecting significant features. Another critical test for metaheuristic algorithms is determining the stability due to the unpredictable and uncertain character of metaheuristic algorithms. Figure 13 compares the stability of the proposed method with other techniques for different tasks and iterations. The strength of agreement of the stability index was divided into three parts: weak, medium, and excellent. If the stability value was less than 0.55 the criteria of value it was considered excellent, or if the result was between 0.56 and 0.69 it was considered medium, and higher than 0.7 it was considered weak. Stability is the algorithm’s capacity to generate similar replies for multiple performances. The five algorithms’ stabilities are depicted in Figure 13 as a function of the number of iterations. It can be seen that the proposed method achieved the best results concerning that parameter. Due to combining the ABC algorithm and GA more problems could be solved compared with the other algorithms.

**Figure 14:**
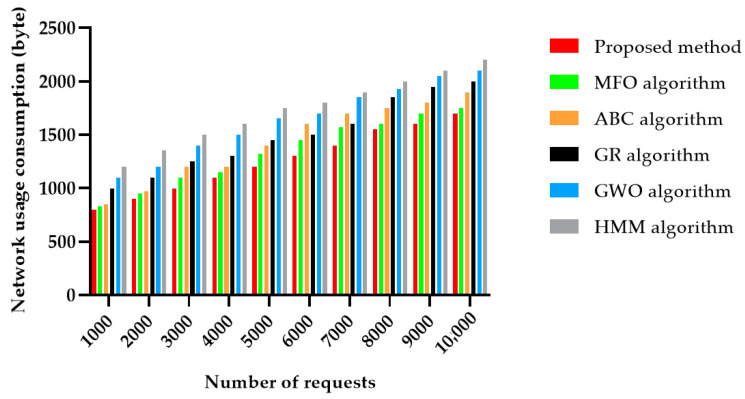
Network usage consumption in 50 different services.

Figure 14 shows the assessment of network resource consumption for all the methods studied, where the 50 services considered different numbers of requests. This figure clearly shows that the proposed algorithm had the lowest network resource usage consumption when compared to the other methods. At the same time, the HMM algorithm was more often used than the different metaheuristic algorithms. Compared with HMM and GWO, our proposed method kept nearly 40 percent, on average, in network resources, regardless of the number of services.

## 5. Conclusions and Future Work

Cloud Computing (CC) has become very popular, due to the benefits offered by cloud services, notably facilities provided by hardware and software and the relatively low cost of the equipment from the user’s point of view. The composition of different services face Np-hard issues, and one single service cannot respond to a large and complex request. These factors determined the necessity of using services composition to develop larger services with superior functionalities. In this paper, GA uses the penalty approach in cases of violation dependency and incompatibility constraints, but this does not mean that the impossible solution is completely eliminated. Impossible solutions help in reaching an achievable solution faster and achieving the customer’s composite service more quickly. In this technique, the GA selects the appropriate services according to user needs. Then, the ABC algorithm evaluates the services selected by GA and combines them, if the services are appropriate. Several experiments were performed with various tasks in Cloud-SIM simulation. The proposed method performed excellently with regards to response time, reliability, and cost, compared to other algorithms. Its energy consumption was higher than the MFO algorithm.

In the future, work could be performed using a neural network in GA. First, the GA performs its typical operations and calculates the chromosomes’ suitability by considering all inter-service relationships and modes. Simultaneously, the chromosome and its degree of suitability are given as input to the neural network algorithm in order to train it during the learning phase. The neural network algorithm predicts the fitness function for the chromosome, and, thus, the GA works normally until the ABC algorithm carries out the combining of the service. A predictable algorithm, such as HMM and Topsis algorithms, can be helpful for service composition in cloud computing as well, and the researchers can use the HMM algorithm to predict the QoS and recommend the appropriate services for users according to their needs.

## Figures and Tables

**Table 1:** The definitions of the QoS parameters for service.

QoS Standards	Measure	Explanation
Response time	ms	The time interval between receiving a demand from one user and answering it.
Energy consumption	j	In the cloud, the machines are running for providing services and these machines also consume energy to perform their tasks.
Cost	$	The expense needed for implementing a certain service.
Availability	Percent	The possibility to access the service from any place at any time.
Reliability	MTBF	The capacity of a certain device (hardware or software) to complete a given task in a specific time, depending on the system requirements.

**Table 2:** Correspondence between GA and service composition.

Definition of the GA	Defining in the Cloud
Chromosome	Abstract services
Generation	Generate new candidate solution
Genome	One candidate solution
Crossover	Different topological services, because the size of services is different.
Parent chromosomes	During evolution, the chromosomes chosen for crossover, according to their fitness values, are known as parents, and the products of crossover are referred to as children.
Fitness function	Evaluate the fitness and goodness of the chromosomes for the problem to be solved.
Mutation	The mutation operator targets at toggle each abstract service in a genome with a probability that may not be found according to user needed.

**Table 3:** Correspondence between ABC algorithm and service composition.

Definition of the ABC	Defining in the Cloud
Food source position	Service composition solution
Food source	Services
Pollen	Quality of Services
New position	New selected service
Previous position	Pervious selected service
Nectar quality	Quality of the composite service
Speed of searching and foraging	Speed of algorithm optimization
The best food source	The optimal service composition solution
Dimension of food source	Dimension of service quality attributes

**Table 4:** Summary of notations used in procedure.

Abbreviations & Parameter	Implication
C	Number of candidate service
¥	Selection space
R	Selection space decrease factor
P	Penalization function
G	Constraint value in case of violation
F	Fitness function
φ	is a random number φ∈[−1, 1]
Priss	A priori service set
SP	A priori exploration strategy
E	Selection space equilibrium selection strategy
[α, β]	The upper and lower limits of the quantity of food supply in each generated set
η	The search step

## Data Availability

Not applicable.
